# Editorial: Trends in Neuroendocrinology

**DOI:** 10.3389/fendo.2016.00041

**Published:** 2016-05-18

**Authors:** Hubert Vaudry

**Affiliations:** ^1^Institut National de la Santé et de la Recherche Médicale (INSERM), Mont-Saint-Aignan, France; ^2^Institute for Research and Innovation in Biomedicine (IRIB), Normandy University, Mont-Saint-Aignan, France; ^3^Laboratory of Neuronal and Neuroendocrine Differentiation and Communication, Rouen University, Mont-Saint-Aignan, France; ^4^International Associated Laboratory Samuel de Champlain, Mont-Saint-Aignan, France

**Keywords:** editorial, neuroendocrinology, ICN2014, oxytocin, circadian rhythm, neuroendocrine factors

**The Editorial on the Research Topic**

**Trends in Neuroendocrinology**

Neuroendocrinology is the field of research that explores the interplay between the central nervous system and the endocrine glands. The neuroendocrine system controls a number of essential physiological processes, including biological rhythms, stress, social behaviors, appetite, growth, and reproduction. The present Research Topic is a compilation of contributions stemming from the 8th International Congress of Neuroendocrinology (ICN2014) held in Sydney, NSW, Australia, that illustrates various facets of current neuroendocrinological investigations.

Most studies on circadian rhythms have been conducted on male animals only, based on the assumption that females display higher variability caused by the interaction of sex hormones with biological rhythms. The review on sex differences in circadian behavioral rhythms by Krizo and Mintz points out the need to include both female and male animals in such studies to elucidate the influence and mechanism of action of gonadal steroids on behavioral rhythmicity. This review also raises the question of the impact of sex hormone changes across the lifespan, notably during the pubertal period, on the circadian system.

The hypothalamo–pituitary–adrenal axis (also called the stress axis) is another fruitful “playground” for neuroendocrinologists. Chen et al. summarize the literature pertaining to the effects of glucocorticoid stress hormones on the nitrergic system notably in the brain. This review clarifies the complex cross-talk between the neuroendocrine stress axis and the nitrergic system that are both implicated in various pathological conditions, including anxiety and depressive disorders. In a sister review, Spiers et al. raise the important question of the effect of glucocorticoids and neuronal oxidative stress. They present the different mechanisms through which cortisol or corticosterone induces oxidative stress, particularly in the hippocampus.

There is now strong evidence that oxytocin influences social behavior in various animal models and even in human. Miller and Caldwell examine the organizational role of oxytocin in the postnatal and peripubertal periods on the brain and behaviors. This review highlights the developmental effects of oxytocin on sexual, affiliative, parental, and aggressive behaviors, as well as on non-social behaviors, such as nociception and addiction. It also investigates the neurochemical systems that mediate the effects of oxytocin on these behaviors.

Given the effects of oxytocin on prosocial behaviors, a role of oxytocin in the etiology and symptom severity of schizophrenia has been hypothesized. In this context, Rich and Caldwell analyze the possible implication of the oxytocin system in the negative symptoms and deficit in social cognition associated with schizophrenia, and discuss its potential for the treatment of schizophrenia.

Reciprocally, early-life adversity and social environment can affect the oxytocinergic system. Alves et al. review the influence of prenatal and postnatal stressors as well as maternal mental health on the development of the oxytocin system in animal models. For studies in human infants, the major challenge is clearly to collect plasma or CSF samples suitable for measurement of oxytocin levels.

Vasopressin, like oxytocin, acts both as a neurohormone released by the neural lobe of the pituitary and as a neurotransmitter/neuromodulator within the brain. Bester-Meredith et al. review the evidence that links vasopressin with the processing of olfactory, auditory, taste, and visual information, and explore how alteration of sensory processing can shape behavioral responses to these stimuli.

The role of neuroendocrine factors in the control of feeding behavior and energy homeostasis has been extensively studied. Thus, the implication of neurotransmitters and neuropeptides in the regulation of the hypothalamic centers that govern appetite and energy expenditure is now relatively well understood (1–7). Méquinion et al. introduce the different animal models that can be used to decipher the physiological, metabolic, and neurobiological alterations associated with anorexia nervosa.

Steroid hormones, including glucocorticoids, mineralocorticoids, androgens, and estrogens, exert their genomic actions through transcription factors known as nuclear receptors. They can also act via membrane receptors that mediate rapid, non-genomic signaling. Rainville et al. describe the various candidates for membrane estrogen and glucocorticoid receptors and focus on the contribution of non-genomic signaling in the control of hypothalamic-driven behaviors by steroid hormones.

Insulin does not only act on liver, muscle, and adipose tissue to regulate glucose homeostasis, but also exerts a central effect on neurophysiological processes. Akintola and van Heemst review the current knowledge on the role of insulin in the central nervous system and the potential implication of insulin signaling in the brain for healthy longevity. The neurotrophin-induced gene VGF encodes a precursor protein that is exclusively expressed in neuronal and neuroendocrine cells. VGF is processed by prohormone convertases to generate a series of biologically active neuropeptides. Lewis et al. describe the various effects of VGF-derived peptides on energy homeostasis, water balance, reproduction, nociception, memory, and learning.

In fish, as in mammals, reproduction is finely regulated by complex neuroendocrine mechanisms. Prasad et al. review the role of serotonin in the control of the reproductive system in teleost fish. Their report provides evidence for coordinated actions of the serotonergic system at different levels of the hypothalamo–pituitary–gonadal axis, supporting the functional significance of serotonin in the control of fish reproduction.

I wish that this Research Topic becomes a major set of references for neuroendocrinologists and raises the interest of other scientists who are not yet working in this fertile domain.

## Author Contributions

The author confirms being the sole contributor of this work and approved it for publication.

## Conflict of Interest Statement

The author declares that the research was conducted in the absence of any commercial or financial relationships that could be construed as a potential conflict of interest.
